# Association between Mediterranean diet and dementia and Alzheimer disease: a systematic review with meta-analysis

**DOI:** 10.1007/s40520-024-02718-6

**Published:** 2024-03-22

**Authors:** Daniele Nucci, Andrea Sommariva, Luca Mario Degoni, Giulia Gallo, Matteo Mancarella, Federica Natarelli, Antonella Savoia, Alessandro Catalini, Roberta Ferranti, Fabrizio Ernesto Pregliasco, Silvana Castaldi, Vincenza Gianfredi

**Affiliations:** 1 Struttura Semplice Dipartimentale Igiene Alimenti E Nutrizione, Dipartimento Di Igiene E Prevenzione Sanitaria, Azienda Di Tutela Della Salute (ATS) Brescia, Via Duca Degli Abruzzi, 15, 25124 Brescia, Italy; 2 Department of Biomedical Sciences for Health, University of Milan, Via Pascal, 36, 20133 Milan, Italy; 3 Department of Biomedical Sciences and Public Health, Università Politecnica Delle Marche, Via Tronto 10/a, 60100 Ancona, Italy; 4 Fondazione IRCCS Ca’ Granda Ospedale Maggiore Policlinico, Via Francesco Sforza 35, 20122 Milan, Italy

**Keywords:** Mediterranean diet, Elderly, Dementia, Alzheimer disease

## Abstract

**Background:**

Dementia affects 5–8% of the population aged over 65 years (~50 million worldwide). Several factors are associated with increased risk, including diet. The Mediterranean diet (MedDiet) has shown potential protective effects against several chronic diseases.

**Aims:**

This systematic review with meta-analysis aim was to assess the association between adherence to the MedDiet and the risk of dementia in the elderly.

**Methods:**

PRISMA-2020 guidelines were followed. PubMed/MEDLINE and Scopus were searched on 17 July 2023. The Newcastle–Ottawa Scale tool was used to assess the risk of bias. The protocol was pre-registered in PROSPERO (registration number: CRD 42023444368). Heterogeneity was assessed using the *I*^2^ test. Publication bias was assessed by visual inspection of the funnel plot and by Egger’s regression asymmetry test. The final effect size was reported as OR or HR, depending on the study design of the included studies.

**Results:**

Out of 682 records, 21 were included in the analysis. The pooled OR was 0.89 (95% CI = 0.84–0.94) based on 65,955 participants (*I*^2^ = 69.94). When only cohort studies were included, HR was 0.84 (95% CI = 0.76–0.94) based on 55,205 participants (*I*^2^ = 89.70). When only Alzheimer Disease was considered OR was 0.73 (95% CI = 0.62–0.85) based on 38,292 participants (*I*^2^ = 63.85).

**Discussion:**

Despite the relatively low risk reduction associated with higher adherence to MedDiet among elderly, it should be considered that this population is the most affected.

**Conclusions:**

Adherence to MedDiet could be an effective non-pharmacological measure to reduce the burden of dementia, even among elderly.

**Supplementary Information:**

The online version contains supplementary material available at 10.1007/s40520-024-02718-6.

## Introduction

Dementia represents a group of several brain degenerative diseases that impair memory, thinking and the ability to perform daily tasks. These diseases are characterized by the destruction of nerve cells and damage to the brain, which in turn leads to a progressive deterioration of cognitive function over time [1]. Dementia can affect people of any age, but it is predominant among the elderly, despite not being a natural part of the aging process. In 2014, approximately 5 million people over the age of 65 lived with dementia, and projections estimate an increase to near to 14 million by 2060 [2]. Moreover, dementia ranks as the 7th leading cause of death and is one of the major causes of disability and dependency in the elderly population, with women suffering the most, especially in terms of higher disability-adjusted life years (DALYs) and mortality [3].

Alzheimer's disease (AD) is the most common cause of dementia, accounting for at least two-thirds of all dementia cases in people over the age of 65 [4]. It is caused by a slowly progressing neurodegenerative accumulation of amyloid-beta peptides (Aβ) which cause neuritic plaques and neurofibrillary tangles [5]. Several factors have been associated with a higher or lower risk of dementia, including Alzheimer’s Disease. Older age, genetic factors, traumatic head injury [6], depression [7], cardiovascular and cerebrovascular disease [8], smoking [9], family history of dementia [10], increased homocysteine levels and Apolipoprotein E (APO-E) ε4 allele have been recognized as potential risk factors [11]. On the contrary, higher education [12], use of anti-inflammatory agents [13], cognitive engagement [14], regular aerobic exercise [14] and healthy diet have been reported to decrease the risk of Alzheimer's disease [4].

Among healthy dietary patterns, the Mediterranean diet has been associated with beneficial effects on several health-related outcomes, including cognitive function [15]. The Mediterranean diet is characterized by a high consumption of whole grains, fruits, vegetables, legumes, and olive oil, with a moderate intake of cheese and fish and a limited intake of meat (especially red and processed meat), sweets and alcohol. Several studies have highlighted the anti-inflammatory effect of the Mediterranean Diet which is considered as one of the main biological pathways through which beneficial effects are mediated [16]. However, results from the literature are not concordant, with some studies reporting that higher adherence to the Mediterranean Diet can improve physical performance and cognitive function [17], delay the onset or prevent dementia and reduce the risk of Alzheimer disease [18], while other studies have not reported any protective effects [19]. Moreover, most of the previous studies refer to adults in general, instead of specifically focusing on the elderly.

Therefore, the primary aim of this systematic review with meta-analysis was to retrieve, collect and collate all the existing evidence in the literature to obtain a comprehensive view on the association between adherence to a Mediterranean diet and risk of dementia among elderly people. Second, this review aims to evaluate critically existing literature, assessing the quality of the studies included and potential biases. Third, using a meta-analytical approach, this review aimed to provide a summary statistical estimation of the strengths of the association between adherence to a Mediterranean diet and dementia, also conducting sensitivity analysis, considering the type of dementia and study design. Lastly, this review also conducted subgroup analyses based on the geographical area and methods used to assess diet.

## Methods

The current systematic review with meta-analysis was conducted according to the guidelines of the Cochrane Collaboration [20], and the results were reported according to the Preferred Reporting Items for Systematic Reviews and Meta-Analyses 2020 (PRISMA-2020) and the Meta-Analysis of Observational Studies in Epidemiology guidelines [21]. The research protocol was defined in advance and shared among the research team. Therefore, the protocol was registered in the international database of prospectively registered systematic reviews (PROSPERO; registration number: CRD 42023444368).

### Literature search strategy

The literature search was conducted simultaneously on PubMed/MEDLINE and Scopus on 17 July 2023, based on the following research question: “Is higher adherence to the Mediterranean diet associated with a lower risk of dementia in the elderly?”. Therefore, the search strategy was developed considering three aspects: the elderly (as population), adherence to the Mediterranean diet (as exposure), and dementia (as outcome of interest). Selected keywords, both MeSH terms and Title/Abstract, were combined using the Boolean operators AND and OR. The search strategy was first developed in PubMed/MEDLINE and therefore adopted for Scopus. The search strategy used for each database is presented in the supplementary Table 1. The searches were performed blindly by two researchers (VG and DN) and an equal number of records were retrieved. Potential additional relevant articles were searched by screening the reference lists of the included articles and consulting experts in the field.

**Table 1:** Main characteristics of included studies, reported in alphabetical order

Author, year	Country	Study period**’**	Study design	Sample size	Attrition	Tool for dietary assessment	Validation	MedDiet score	Dementia type	Diagnostic assessment
Allcock, 2022	Australia	2022	Cross-sectional	294	9 (2.9%)	14 items MEDAS	Yes	14-item MEDAS	All type of dementia	AD8
Anastasiou CA, 2017	Greece	n.a.	Cross-sectional	1864	n.a.	69 items FFQ	Yes	MedDietScore, proposed by Panagiotakos et al.	AD, vascular dementia, Lewy body and frontotemporal dementias	Semi-structured interview of the CDR
Calil Silvia RB, 2018	Brazil	n.a.	Cross-sectional	96	0	98 items FFQ	n.a.	MedDietScore, proposed by Panagiotakos et al.	MCI and AD	Clinical assessments performed by a geriatrician or neurologist
Chan R, 2013	Hong Kong	2001–2003	Cross-sectional	3670	330 (8.2%)	280 items FFQ	Yes	MedDietScore, proposed by Trichopoulou et al.	Cognitive impairment	CSI-D
Charisis, 2021	Greece	n.a.; 3.1 ± 0.9 years of FU	Cohort	1046	2.4%	69 items FFQ	Yes	MedDietScore, proposed by Panagiotakos et al.	AD, VaD, Lewy body and frontotemporal dementias	CDR
de Crom TO, 2022	Netherlands	I sub-cohort 1989–1993 baseline II sub-cohort 2009–2012 baseline. No data on FU	Cohort	I sub-cohort 5375 II sub-cohort 2861	I sub-cohort: 32.7% II sub-cohort: 5%	I sub-cohort: 170 items FFQ II sub-cohort: 389 items FFQ	Yes	MedDietScore, proposed by Panagiotakos et al.	All-type of dementia	Clinical assessment based on DSM-III-R and NINCDS-ADRDA for AD
Féart, 2009	France	2001–2007	Cohort	1410	1213 (14%)	FFQ and 24 h recall	Yes	MedDietScore, proposed by Trichopoulou et al.	Dementia and AD	Battery of neuropsychological tests + neurological evaluation
Gardener, 2012	Australia	n.a.	Cross-sectional	970	n.a.	74 items CCVFFQ	Yes	MedDietScore, proposed by Trichopoulou et al.	MCI and AD	Clinical assessment based on diagnostic criteria DSM-IV and ICD-10
Glans I, 2023	Sweden	1991–2014; 23 years of FU	Cohort	28,025	2421 (7.9%)	7-day food diary, detailed FFQ, and 1-h interview	n.a.	mMDS	All-type dementia, AD, VaD	Registered dementia diagnoses from the Swedish National Patient Register (Inpatient + hospital)
Gu Y, 2010	USA	1992–2008; 3.8 year of FU	Cohort	1219	1559 (56.1%)	61 items version of Willett’s FFQ	Yes	MedDietScore, proposed by Trichopoulou et al.	AD	DSM-III-R and criteria of the NINCDS-ADRDA
Haring, 2016	USA	1996–1999; 9.11 years of FU	Cohort	6425	1054 (14.1%)	WHI FFQ	Yes	aMDS	Probable dementia	Functional test + clinical evaluation + noncontrast CT brain scan and laboratory blood tests
Hosking DE, 2019	Australia	2001–2014; 12 years of FU	Cohort	1220	1331 (52.2%)	CSIRO semi-quantitative FFQ	Yes	MedDietScore, proposed by Trichopoulou et al. and proposed by Panagiotakos et al.	AD and MCI (combined)	Neuropsychological testing and MMSE
Mamalaki E, 2022	Greece	n.a.	Cohort	1018	n.a.	69 items FFQ	Yes	MedDietScore, proposed by Panagiotakos et al.	All-type of dementia	Multiple neuropsychological validated tests and clinical assessment
Morris, 2015	USA	2004–2013; 9 years of FU	Cohort	923	40.26%	Modified version of the Harvard semi-quantitative FFQ	Yes	MedDietScore, proposed by Panagiotakos et al.	AD	Neurological examination, medical history and cognitive performance tests
Nicoli, 2021	Italy	2003–2018; 12 years FU	Cross-sectional and cohort	Cross-sectional = 1390; cohort = 512	76.7%	FFQ	Yes	MDS*	All-type of dementia	Clinical assessment based on DSM-IV, and MMSE
Olsson, 2014	Sweden	1970–1994; 12 years FU	Cohort	1038	55%	Optically readable form of a 7-day food record based on a validated pre-coded menu book	Yes	mMDS	AD and all-type dementia	Clinical assesment and MMSE
Roberts, 2010	USA	2004; 2.2 years OF fu	Cohort	1233	736 (37.4%)	128 items modified Block 1995 Revision of the HHH Questionnaire	Yes	MedDietScore, proposed by Trichopoulou et al.	MCI and dementia	According to the DSM-IV criteria
Scarmeas, 2006a	USA	1992–1999	Case–control study nested within a community-based cohort	1984: 194 patients with AD vs 1790 nondemented subjects	52%	61 items Willett’s semiquantitative FFQ	Yes	MedDietScore, proposed by Trichopoulou et al.	AD	CDR and clinical assessment. Alzheimer disease was categorized based on NINCDS-ADRDA
Scarmeas, 2006b	USA	Baseline 1992–1999; 4 ± 3 years of FU	Cohort	2258	1908 (45%)	61 items Willett’s semiquantitative FFQ	Yes	MedDietScore, proposed by Trichopoulou et al.	AD	CDR and clinical assessment. Alzheimer disease was categorized based on NINCDS-ADRDA
Scarmeas, 2009	USA	Baseline 1992–1999; 10 years of FU	Cohort	1875	1818 (49%)	61 items FFQ	Yes	MedDietScore, proposed by Trichopoulou et al.	MCI and AD	Clinical assessment
Talhaoui, A, 2023	Morocco	March 2017–May 2018	Cross-sectional	151	86 (36.3%)	Questionnaire of weekly food consumption	Yes	MedDietScore, proposed by Panagiotakos et al.	Cognitive impairment	MMSE

**’**For cohort studies, the duration of follow-up was also reported

*AD* Alzheimer's Disease, *AD8* dementia screening intervention, *aMDS* alternate Mediterranean diet score, *CCVFFQ* Cancer Council of Victoria Food Frequency Questionnaire, *CDR* Clinical Dementia Rating Scale, *CSI-D* Community Screening Instrument for Dementia, *CSIRO* Commonwealth Scientific and Industrial Research Organization, *CT* computed tomography; *HHH* Health Habits and History, *DSM-III-R* Diagnostic and Statistical Manual of Mental Disorders 3rd edition, *DSM-IV* Diagnostic and Statistical Manual of Mental Disorders 4th edition, *FFQ* food frequency questionnaire, *FU* follow-up, *ICD-10* International Classification diseases version 10, *MCI* mild cognitive impairment, MDS* Mediterranean diet score calculated as the sum of intakes of nine dietary components: high intake of cereals, fruits, vegetables, legumes, fish, monounsaturated/saturated fat ratio, low intake of milk and meat, and moderate alcohol intake, *MEDAS* Mediterranean Diet Adherence Screener, *MedDietScore* Mediterranean Dietary Score, *mMDS* modified Mediterranean diet score, *MMSE* Mini-mental state examination, *NINCDS-ADRDA* National Institute of Neurological and Communicative Disorders and Stroke and the Alzheimer's Disease and Related Disorders Association, *VaD* vascular dementia, *WHI* Women’s Health Initiative

### Inclusion/exclusion criteria

Inclusion/exclusion criteria were defined according to the following guidelines: Population (P), Exposure (E), Comparison (C), Outcome (O), Study design (S). In particular, only observational epidemiological studies in elderly people (over 60 years of age), assessing the association between adherence to the Mediterranean diet and dementia (any type), published in English in an international, peer-reviewed scientific journal, were considered eligible. In contrast, non-original or interventional studies assessing the association between adherence to any other type of diet and a health outcome other than dementia in people younger than 60 years, not published in English and not in a peer-reviewed journal were excluded. A detailed description of the inclusion/exclusion criteria, defined according to PECOS, is provided in the supplementary Table 2.

**Table 2:** Main characteristics of studied population

Author, year	Population characteristics	Age	Sex	Effect size (95% CI) *p*-value	Adjustments
Allcock, 2022	Community-dwelling older adults	70.4 ± 6.2	F = 205 (67.7%)	Beta = −0.134 (−0.198, −0.007) 0.035	Age, gender, BMI, smoking status, average sleep duration/night, average physical activity duration/day, diabetes status and education status
Anastasiou CA, 2017	Community-dwelling adults aged ≥ 65 years old from HELIAD	73.0 ± 6.1	M = 757; F = 1107	OR = 0.92 (0.87–0.97) 0.004	Age, sex, education, number of clinical co-morbidities, energy intake
Calil Silvia RB, 2018	Adults over the age of 60 years recruited and assessed at the Paulista IPGG and in the Neurology Outpatient clinic of the Santa Marcelina Hospital	≥ 60	M = 27; F = 69	n.a.	n.a.
Chan R, 2013	Adults aged ≥ 65 years old of a cohort study examining the risk factors for osteoporosis, volunteers and able to walk or take public transport to the study site	≥65	M = 1926; F = 1744	M: OR = 0.89 (0.56–1.41) 0.925 F: OR = 1.02 (0.75–1.41) 0.952	Age, BMI, PA, energy intake, education level, Hong Kong ladder, community ladder, smoking status, alcohol use, activities of Daily Living, depression, self-reported history of DM, hypertension, CVD/ stroke
Charisis, 2021	Community-dwelling adults aged ≥ 65 years old from HELIAD	73.1 ± 5.0	M = 40.3%	HR 0.10 (0.01–0.78) 0.029	Age, sex, years of education, energy intake, BMI, number of clinical comorbidities, MCI at baseline, center of evaluation, PA, Apo E genotype
de Crom TO, 2022	The first two subcohorts of the Rotterdam Study (RS), among inhabitants from the suburb Ommoord in Rotterdam	RS-I 67.7 ± 7.8 and RS-II 75.3 ± 5.9	F = 59.0%	I sub-cohort ( 1989–993) N 1188/5375 HR 1.04 (0.97–1.10)II sub-cohort( 2009–2012) N 248/2861 HR = 0.75 (0.66–0.86)	Sex, age, educational level, smoking status, physical activity, and energy intake, BMI, diabetes, hypercholesterolemia, and hypertension
Féart, 2009	Community dwellers from Bordeaux, France, included in The Three-City (3C) study	75.9	M = 527; F = 883	Dementia HR 0.92 (0.51–1.66) *p* = 0.78; Alzheimer's disease HR 0.70 (0.34–1.43) *p* 0.33	Sex, education, marital status, total energy intake, practice of physical exercise, taking ≥ 5 drugs/d, Center for Epidemiological Studies-Depression scale score, Apolipoprotein E genotype, BMI, hypertension, hypercholesterolemia, diabetes, tobacco, stroke
Gardener, 2012	Adults over the age of 60 years	71.72 ± 7.86	M = 407; F = 563	MCI OR 0.87 (0.75–1.00) *p* < 0.05AD OR 0.81 (0.71–0.92) *p* < 0.01	Age, sex, country of birth, education, apolipoprotein E allele status, current smoking status, caloric intake, BMI, history of stroke, diabetes, hypertension, angina and heart attack
Glans I, 2023	Nondemented individuals born 1923–1950 and living in Malmo (Malmo Diet and Cancer study)	≥ 60	F = 16,992; M = 11,033	All-cause dementia HR 0.95 (0.76–1.18) AD HR 0.87 (0.66–1.16)	Sex, age, education, dietary assessment method, season and total calorie intake, smoking, physical activity, alcohol consumption and BMI
Gu Y, 2010	Medicare-eligible northern Manhattan residents	76.7 ± 6.4	F = 812; M = 407	HR 0.87 (0.78–0.97)	Age, gender, race, education, fasting insulin, adiponectin level, high-sensitivity C-reactive protein
Haring, 2016	Postmenopausal women enrolled in the WHIMS study	65–79	F = 6425	HR 1.13 (0.79, 1.63)	Age, race, education level, WHI Hormone Trial Randomization assignment (HTR arm), baseline 3MSE level, smoking status, physical activity, diabetes status, hypertension status, BMI, family income, depression, history of CVD and total energy intake
Hosking DE, 2019	PATH through life study	62.5 ± 1.5	n.a.	Using MedDietScore by Trichopoulou OR = 0.77 (0.43–1.39) Using MedDietScore by Panagiotakos et al. (1.30 (0.79–2.15)	Energy intake, age, sex, and APOE3 allel 4, education, mental activity, physical activity, smoking status, depression, diabetes, BMI, hypertension, heart disease, and stroke
Mamalaki, 2022	Community-dwelling adults aged ≥ 65 years old from HELIAD	73.1 ± 5.0	F = 59.7%	RR 0.968 (0.955–0.982) < 0.001	Age, sex and years of education
Morris, 2015	Volunteers living in retirement communities and senior public housing units in the Chicago area enrolled in the Rush MAP	81	M and F (no data)	HR 0.46 (0.29, 0.74) *p* < 0.001	Age, sex, education, APOE-ε4, total energy intake, physical activity and participation in cognitively stimulating activities
Nicoli, 2021	Adults aged ≥ 80 years old residents in the province of Varese	93.2	F = 73.0%	OR = 0.97 (0.71–1.31) *p* = 0.008; HR = 1.17 (0.82–1.66) *p* = 0.369	Age, sex, education, total intake of kilocalories, smoke, alcohol, physical activity, hypertension, chronic obstructive pulmonary disease, diabetes, lifetime depression, previous stroke, previous transient ischemic attack
Olsson, 2014	Men born in 1920–24 and living in Uppsala	71 ± 0.6	M	AD: HR = 0.99 (0.44–2.26) *p* = 0.95; dementia HR = 0.84 (0.46–1.53) *p* = 0.69	Energy, educational level, apolipoprotein E (APOE) genotype and PA
Roberts, 2010	Residents from Olmsted County, USA, aged 70–89 years	70–89	F = 592	OR 0.78 (0.51–1.22) *p* = 0.28	Age, years of education, total energy (continuous variables), sex, ApoE ε4 (ε4+ vs. ε4−), stroke, coronary heart disease, and depressive symptoms
Scarmeas, 2006a	Medicare beneficiaries residing in Manhattan	76.3 ± 6.6	M = 630; F = 1354	OR 0.31 (0.16–0.58) *p* < 0.001	Cohort, age at intake in the study, sex, ethnicity, education, APOE genotype, smoking, comorbidity index, and BMI
Scarmeas, 2006b	Medicare beneficiaries residing in Manhattan	77.2 ± 6.6	M = 720 (32%); F = 1538 (68%)	HR 0.60 (0.42–0.87)	Cohort, age, sex, ethnicity, education, apolipoprotein E genotype, caloric intake, smoking, comorbidity index, and body mass index
Scarmeas, 2009	Medicare beneficiaries residing in Manhattan	76.9 ± 6.5	M = 603 (32%); F = 1272 (68%)	AD: HR = 0.52 (0.30–0.91); *p* = 0.02; MCI: HR = 0.72 (0.52–1.00) *p* = 0.05	Cohort, age at intake in the study, gender, ethnicity, education, APOE, BMI and time between 1st dietary and 1st cognitive status assessment
Talhaoui A, 2023	Moroccan elderly subjects from three nursing homes	≥60	F = 61 (40.45)	OR = 0.96 (0.85–1.08) *p* > 0.05	Gender, profession, pension, education, nutritional status, Fat-Free-masse, calf circumference, GDS-15 score, and walking or cycling PA

*AD* Alzheimer disease, *F* female, *HELIAD* Hellenic Longitudinal Investigation of Aging and Diet, *HR* Hazard ratio, *IPGG* Institute of Geriatrics and Gerontology Jose Ermírio de Moraes, *M* male, *MAP* Memory and Aging Project, *MCI* Mild Cognitive Impairment, *OR* Odds ratio, *PATH* Personality and Total Health, *WHIMS* Women’s Health Initiative Memory Study

### Study selection and data extraction

As previously done [22, 23], the selection of studies was carried out in two stages. First, titles and abstracts of records retrieved using the search strategy and those retrieved from additional sources were screened independently by two reviewers using the inclusion/exclusion criteria above. Secondly, the full-text was searched and downloaded only for potentially eligible articles. These were then assessed independently by two reviewers. Any disagreements about the eligibility and inclusion of articles were resolved by discussion between the reviewers. If disagreement persisted, a third senior researcher was involved to make the final decision. The extracted data were collected using a standardized, and pre-defined spreadsheet using Excel (Microsoft Excel® for Microsoft 365 MSO, USA, 2019). To improve the quality of data extraction, the spreadsheet was pre-tested on five randomly selected studies. The following information was extracted from each included study: first author, year of publication, the country in which the study was conducted, study period, study design, number of participants, age and sex, main population characteristics, number of participants lost (attrition rate), dietary assessment tool used, whether or not the tool was validated, Mediterranean diet score used to assess adherence, diagnostic tool used to diagnose dementia, type of dementia, maxim adjusted effect size measurements along with the corresponding 95% CIs, variables used for adjustment, whether funding was received for conducting the original study, and declared conflicts of interest. Data extraction was performed in duplicate and discrepancies were resolved by discussion. Missing data were obtained by contacting the corresponding author.

### Data synthesis

Following the PRIMA 2020 guidelines, the selection process was documented using a “flow diagram” showing the number of references excluded at each step. Reasons for study exclusion after full-text assessment are reported in detail. In addition, the extracted data were tabulated and summarized in text. Moreover, the results of the statistical analysis are presented in both tables and figures (detailed below).

### Quality assessment

The Newcastle–Ottawa Scale (NOS) was used to assess the methodological quality and risk of bias of the included studies. The scale is based on a 'star system' in which studies are graded on three main aspects: the selection of study groups, the comparability of the groups, and the ascertainment of either the exposure or the outcome of interest. The overall quality score was considered as a continuous variable; however, taking into account the previously adopted cut-off, the studies were considered to be of high quality if the NOS score was equal to or greater than 7 points.

### Statistical analysis

Data were pooled using a meta-analysis focusing on the overall association between higher adherence to the Mediterranean diet and the risk of any type of dementia (including mild cognitive impairment). The summary effect size was calculated based on the odds ratio (OR), hazard ratio (HR), and risk ratio (RR) of the included studies. The final effect size was reported as OR or HR based on the study design of the included studies. In particular, an OR was reported in the subgroup analysis that included only cross-sectional studies. Conversely, in the subgroup that included only longitudinal studies, the effect size was calculated using HR. In the current meta-analysis, random and fixed effect models were used. Heterogeneity was assessed using the *I*^2^ test, which measures the proportion of total variance between studies that is beyond random error. Based on the results obtained, heterogeneity was classified as high when *I*^2^ values were equal to or greater than 75%, moderate when *I*^2^ values were between 50 and 75%, low when *I*^2^ values were between 25 and 50%, and finally, no heterogeneity when *I*^2^ values were equal to or less than 25%. Publication bias was assessed by both visual inspection of the funnel plot and by Egger’s regression asymmetry test, with statistical significance set at *p* < 0.10. If publication bias was detected, the trim and fill method was used to adjust for it by searching for missing studies to the right of the total. All data analyses were performed using Prometa3® software (Internovi, Cesena, Italy).

### Sensitivity analyses

A sensitivity analysis was conducted based on type of dementia (including only unspecified dementia and mild cognitive impairment; only unspecified dementia; only Alzheimer disease; only mild cognitive impairment). Moreover, a sensitivity analysis was performed that included only studies of high methodological quality. Finally, studies based on the same population were excluded to avoid potential overlapping effects. In this case, only studies with the highest NOS score or, in case of a tie, the study with the larger sample size were selected.

### Subgroup analyses

Subgroup analysis was performed by study design, country in which the study took place (including only studies conducted in Mediterranean countries), sex, and only including studies that used validated tools. Subgroup analyses were only performed when three or more studies were available.

## Results

### Literature search

A total of 682 records were identified by searching Pubmed/MEDLINE (*n* = 257) and Scopus (*n* = 425). No additional articles were included based on reference screening and expert consultation.

**Fig. 1:**
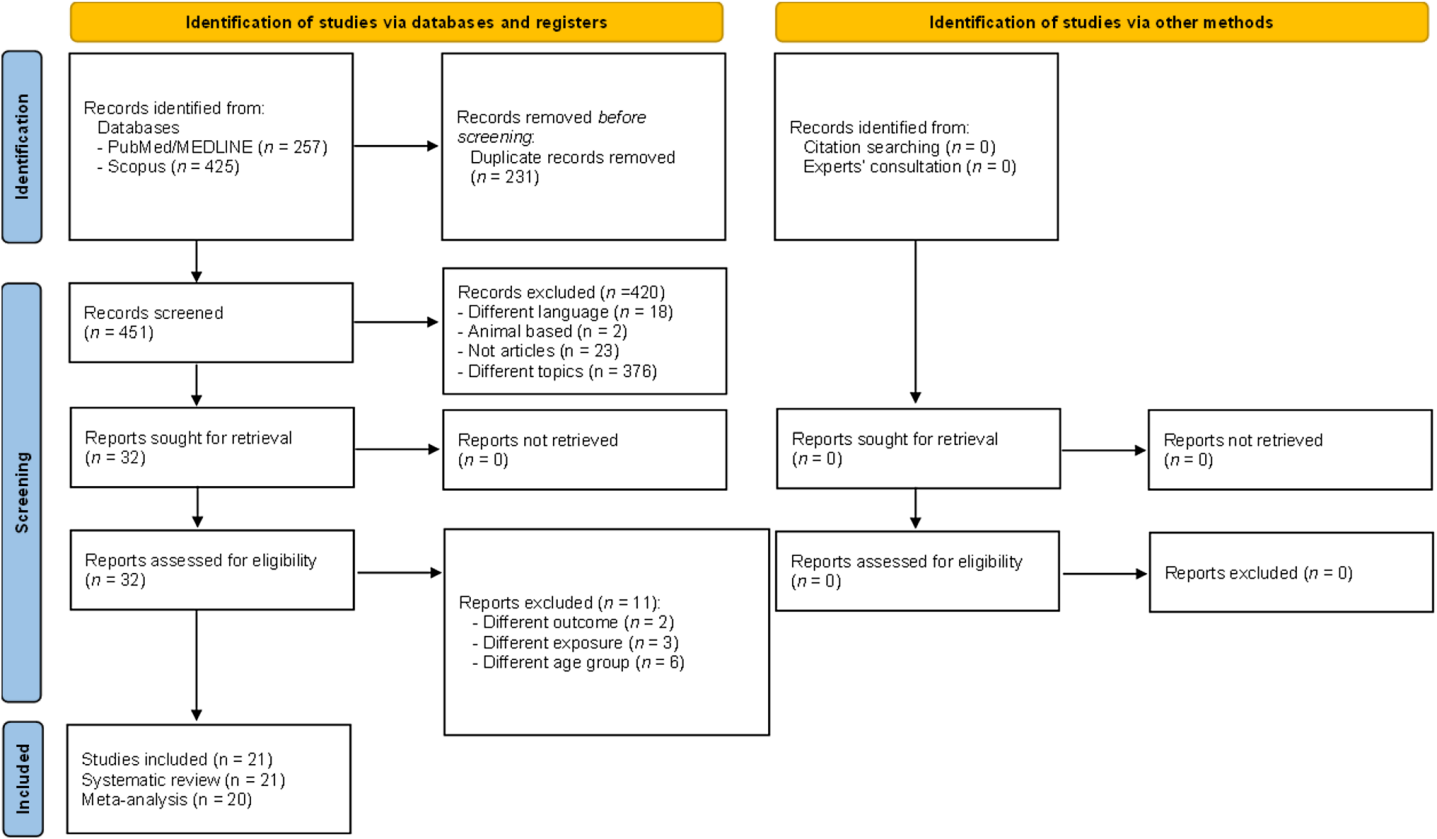
PRISMA flow diagram showing the selection process

After preliminary exclusion of duplicates (*n* = 231), a total of 451 records were screened based on title and abstract. Based on the initial screening, 420 records were removed due to different language (*n* = 18), non-human studies (*n* = 2), non-original work (*n* = 23) and focus on different topics (*n* = 376), leaving 32 records eligible for inclusion. Based on full-text assessment, 11 records were excluded (reasons for exclusion are detailed in the supplementary Table 3) [24–34], resulting in 21 records included in the current systematic review [19, 35–54]; however, one record did not provide analytical data and, therefore, could not be included in the meta-analysis [37]. The selection process is shown in Fig. 1.

**Table 3:** Summary statistics of the main, sensitivity and subgroup analyses

	Summary statistics					Publication bias	
Analysis	Studies included [Ref.]	No. of participants	df	ES (95% CI); *p*-value	*I*^2^; *p*-value	Intercept’; *p*-value	Estimated^a^ ES; *p*-value
All type of dementia: including unspecified dementia, Alzheimer disease and mild cognitive impairment							
All type of dementia^b^	[19, 35, 36, 38–54]	65,955	20	OR^ = 0.96 (0.95–0.97); <0.001	69.55; <0.001	−1.19; 0.005	OR^ = 0.97 (0.95–0.98); <0.001
				OR” = 0.89 (0.84–0.94); <0.001			OR” = 0.95 (0.89–1.02); 0.145
All type of dementia^c^	[19, 35, 36, 38–54]	65,955	20	OR^ = 0.96 (0.95–0.97); <0.001	69.94; <0.001	−1.08; 0.013	OR^ = 0.97 (0.95–0.98); <0.001
				OR” = 0.89 (0.84–0.95); <0.001			OR” = 0.92 (0.86–0.99); 0.019
All type of dementia cross-sectional	[36, 38, 41, 48, 51, 54]	10,029	6	OR^ = 0.92 (0.88–0.96); <0.001	52.25; 0.050	−0.78; 0.394	n.a.
				OR” = 0.91 (0.82–1.00); 0.055			
All type of dementia cohort studies	[19, 39, 40, 42–50, 52, 53]	55,205	14	HR^ = 0.97 (0.96–0.99); 0.001	89.70; <0.001	−1.76; 0.053	HR^ = 0.99 (0.97–1.00); 0.039
				HR” = 0.84 (0.76–0.94); 0.002			HR” = 0.85 (0.76–0.95); 0.003
Dementia: including diagnosis of unspecified Dementia and/or mild cognitive impairment							
Dementia^b^	[36, 38–42, 44–46, 48–50, 52, 54]	59,571	16	OR^ = 0.96 (0.95–0.98); <0.001	53.56, 0.005	−0.72; 0.078	OR^ = 0.97 (0.96–0.98); <0.001
				OR” = 0.93 (0.88–0.98); 0.005			OR” = 0.96 (0.91–1.02); 0.196
Dementia^c^	[19, 36, 38–42, 44–46, 48–50, 52, 54]	59,571	16	OR^ = 0.96 (0.95–0.98); <0.001	54.62; 0.004	−0.58; 0.170	OR^ = 0.97 (0.96–0.98); <0.001
				OR” = 0.93 (0.89–0.98); 0.009			OR” = 0.94 (0.89–0.99); 0.024
Dementia cross sectional	[36, 38, 41, 48, 54]	8045	5	OR^ = 0.92 (0.88–0.97); <0.001	0.00; 0.903	0.21; 0.631	n.a.
				OR” = 0.92 (0.88–0.97); <0.001			
Dementia cohort studies	[19, 39, 40, 42, 44–46, 48–50, 52]	50,805	11	HR^ = 0.98 (0.97–1.00); 0.025	63.07; 0.002	−0.86; 0.113	HR^ = 0.99 (0.98–1.00); 0.025
				HR” = 0.94 (0.87–1.01); 0.083			HR” = 1.00 (0.92–1.08); 0.912
Dementia: only including diagnosis of unspecified Dementia							
Dementia	[19, 36, 38–41, 44, 46, 48, 49]	55,092	12	OR^ = 0.96 (0.95–0.98); <0.001	59.86; 0.003	−0.62; 0.239	OR^ = 0.97 (0.95–0.98); <0.001
				OR” = 0.94 (0.88–0.99); 0.021			OR” = 0.94 (0.88–0.99); 0.021
Dementia cross sectional	[36, 38, 41, 44, 48]	14,319	5	OR^ = 0.92 (0.88–0.97); <0.001	0.00; 0.797	0.41; 0.379	n.a.
				OR” = 0.92 (0.88–0.97); <0.001			
Dementia cohort studies	[19, 39, 40, 42, 46, 48, 49]	41,285	7	OR^ = 0.99 (0.97–1.00); =0.033	70.74; 0.001	−0.91; 0.273	OR^ = 0.99 (0.98–1.00); =0.033
				OR” = 0.95 (0.88–1.03); =0.196			OR” = 0.99 (0.91–1.09); 0.900
Only including diagnosis of Alzheimer’s disease							
Overall Alzheimer's disease	[41–43, 47, 49, 51–53]	38,292	7	OR^ = 0.81 (0.75–0.87); <0.001	63.85; 0.007	−1.88; 0.055	OR^ = 0.83 (0.77–0.89); <0.001
				OR” = 0.73 (0.62–0.85); <0.001			OR” = 0.76 (0.63–0.90); 0.002
Alzheimer’s disease (cohort studies)	[42, 43, 47, 49, 52, 53]	34,300	5	HR^ = 0.79 (0.75–0.84); <0.001	91.78; <0.001	−1.57; 0.629	HR^ = 0.80 (0.76–0.85); <0.001
				HR” = 0.73 (0.58–0.93); 0.010			HR” = 0.82 (0.66–1.02); 0.075
Only including diagnosis of mild cognitive impairment							
Mild cognitive impairement (all)	[41, 52, 54]	2996	2	OR^ = 0.91 (0.83–0.99); =0.028	35.49; 0.212	−2.72; 0.259	OR^ = 0.96 (0.89–1.03); =0.276
				OR” = 0.89 (0.79–1.01); 0.063			OR” = 0.96 (0.85–01.09); 0.525
Mediterranean area	[19, 36, 39, 46, 48, 54]	6879	5	OR^ = 0.96 (0.95–0.98); <0.001	31.91; 0.196	−0.86: 0.171	OR^ = 0.97 (0.96–0.98); <0.001
				OR” = 0.95 (0.91–0.99); 0.021			OR” = 0.97 (0.92–1.01); 0.142
Validated exposure	[19, 35, 36, 38–41, 43–54]	37,930	19	OR^ = 0.96 (0.95–0.97); <0.001	71.07; <0.001	−1.24; 0.005	OR^ = 0.97 (0.95–0.98); <0.001
				OR” = 0.89 (0.83–0.94); <0.001			OR” = 0.95 (0.89–1.02); 0.126

^a^Estimated using the trim and fill analysis

^b^Hosking et al. assessed adherence to the Mediterranean diet using two different scores, therefore, in this analysis effect size using Trichopoulou’s score was used

^c^Hosking et al. assessed adherence to the Mediterranean diet using two different scores, therefore, in this analysis effect size using Panagiotakos’ score was used

’Calculated using Egger’s linear regression test, ^fixed effects model;” random effects model

*CI* confident interval, *df* degree of freedom, *ES* effect size, *n.a.* not applicable, *OR* odds ratio, *HR* hazard ratio

### Main characteristics of included studies

Almost all continents were represented with Europe with eight studies (Greece *n* = 3 [36, 39, 46], Sweden *n* = 2 [42, 49], Netherlands *n* = 1 [40], France *n* = 1 [19], Italy *n* = 1 [48]), followed by the United States of America with seven studies [43, 44, 47, 50–53], three studies were conducted in Australia [35, 41, 45], one study was conducted in Brazil [37], one study in Hong Kong [38], and one study in Morocco [54]. Regarding the study period, the first cohort was established in 1970 [49], while the most recent study was conducted in 2022 [35]. In terms of study design, half of the included studies were cohort studies (*n* = 13) [19, 39, 40, 42–47, 49, 50, 52, 53], followed by cross-sectional studies (*n* = 6) [35–38, 41, 54], one study performed both cross-sectional and longitudinal analysis [48], and lastly one study is a case–control study nested within a cohort study [51]. Sample sizes ranged from 96 [37] to 28,025 participants [42]. The attrition rate (based on non-compliance or loss to follow-up) ranged from 0 [37] to 76.7% [48]; however, three studies did not report this information [36, 41, 46]. Dietary assessment was mainly based on food frequency questionnaires (FFQ), with the number of items ranging from 14 [35] to 389 [40]. Only two studies used a combination of FFQ and 24-h recall [19] or a 7-day food diary [42], while one study used a 7-day food diary [49]. All questionnaires used were validated, but two studies did not report this information [37, 42]. Adherence to the Mediterranean diet was estimated using different types of scores. In particular, eight studies used the score proposed by Trichopoulou et al. [19, 38, 41, 43, 50–53]; seven studies used the score proposed by Panagiotakos et al. [36, 37, 39, 40, 46, 47, 54], one study used both scores [45], two studies used the modified Mediterranean Diet Score (mMDS) [42, 49], one study used the alternate Mediterranean Diet Score (aMDS) [44], one study used the 14-item Mediterranean Diet Adherence Screener (MEDAS) [35], and one study calculated adherence using defined self-defined score [48]. Details are given in Table 1.

### Main characteristics of the study population

Recruited participants were all over 60 years of age (due to our inclusion criteria) with the oldest population being 93 years of age [48]. In the majority of included studies, participants were community-dwelling; however, four studies only included Medicare beneficiaries [43, 51–53] and three studies included participants from healthcare institutions (neurology outpatients [37], retirement communities [47], and nursing homes [54]). All studies included both women and men, but one study included only postmenopausal women [44], and one study included only men [49]. More details are given in Table 2.

### Quality assessment

All included studies scored 7 or higher and were therefore considered to be of high quality. Only the study not included in meta-analysis was considered as moderate quality (main reasons were attributable to the statistical analysis). Approximately half of the included studies (*n* = 12) reported no conflicts of interest, five studies did not report this information [43, 44, 51–53], while four studies reported conflicts of interest [47–49, 54]. However, 15 studies received funding to conduct the research, four studies did not report this information [43, 49, 52, 53], and finally two studies did not receive funding [19, 54]. Detailed quality assessment, reported item by item, is described in Supplementary Table 4. Inter-rater reliability was assessed and the discrepancy between the two reviewers was approximately 5%. Disagreements were resolved by discussion, and final agreement was reached for all included studies.

### Meta-analysis: MedDiet adherence and all type of dementia

As one study reported results separately for males and females [38], and one study reported data separately for the two included cohorts [40], they were considered to be independent studies. Finally, one study did not report quantitative data [37], and another reported data using the beta coefficient [35] (which is not statistically comparable with all other risk estimates collected), and for these reasons they were excluded from the meta-analysis. Therefore, a total of 21 data sets were included in the main analysis.

**Fig. 2:**
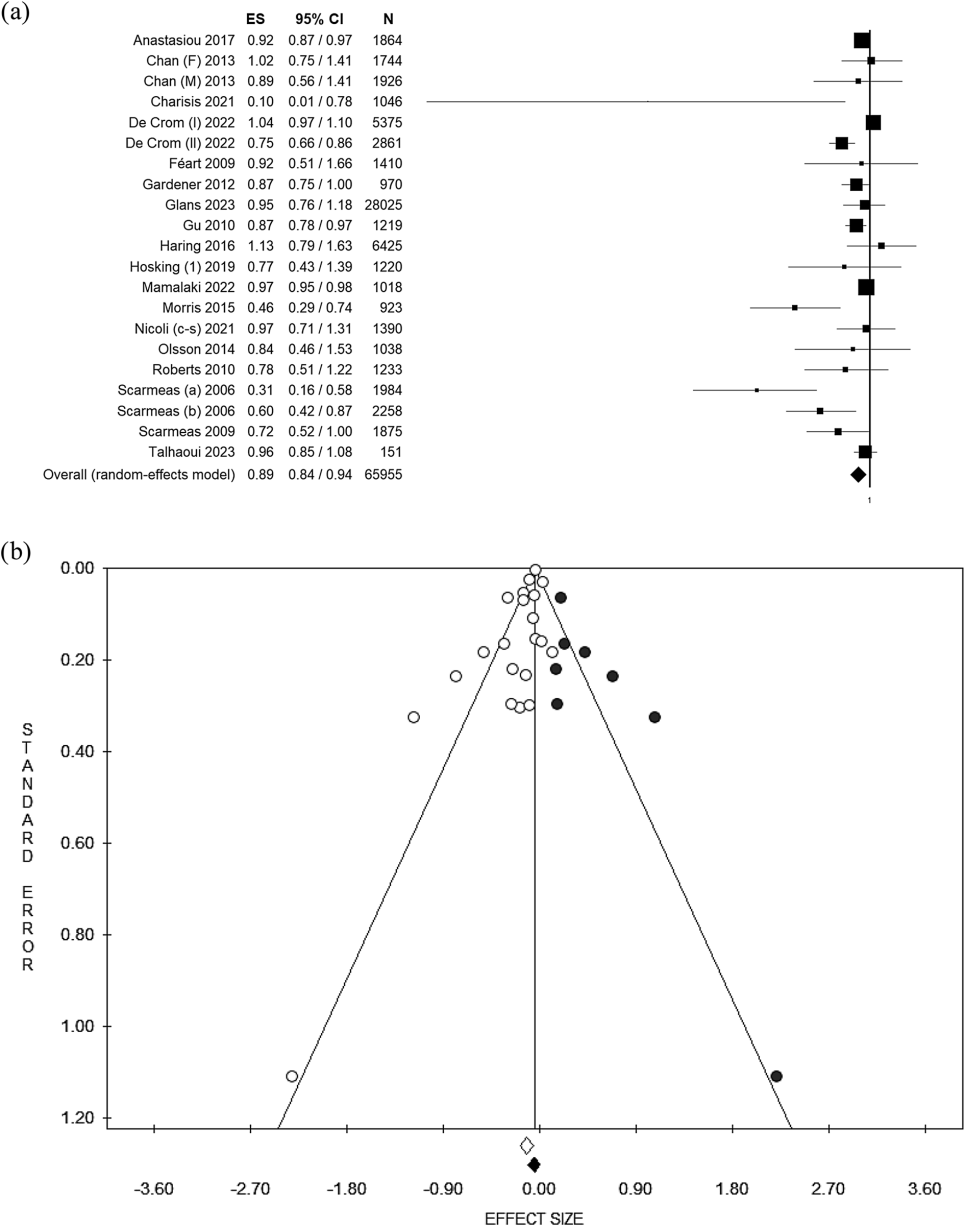
Forest plot (**a**) and Funnel plot (**b**) using the random effect model of the main analysis. The *white dots* represent the included studies. The *white diamond* represents the calculated Effect size. The *black dots* represent the estimated studies after the trim and fill method. The *black diamond* represents the estimated ES after the trim and fill method. *ES* effect size, *95% CI* 95% confidence interval

Considering all 21 data sets and using the random effect model, the pooled OR was 0.89 [(95% CI = 0.84–0.94); *p*-value < 0.001] based on 65,955 participants (Fig. 2a) with moderate statistical heterogeneity (df = 20, *I*^2^ = 69.94, *p*-value ≤ 0.001). Potential publication bias was identified by visual assessment of the funnel plot (Fig. 2b) and confirmed by Egger’s linear regression test (intercept −1.08, *p*-value = 0.013). After applying the trim and fill method, the estimated effect sizes were not significantly different from the main result. Given that one study estimated the adherence to the MedDiet using two different scores [45], and given the heterogeneity of the MedDiet scoring systems used in all included studies, and to improve the consistency and comparability between studies, we decided to perform an additional analysis alternatively pooling the two scores. However, the results did not change. The results for both the fixed and random effect models are shown in Table 3.

### Sensitivity analyses

Sensitivity analyses were performed based on the type of dementia. In particular, the pooled effect size for the risk of dementia, including only unspecified dementia and mild cognitive impairment, was calculated based on 17 studies. Using the random effect model, the pooled OR was 0.93 [(95% CI = 0.88–0.98); *p*-value = 0.005] based on 59,571 participants with moderate statistical heterogeneity (df = 16, *I*^2^ = 53.56, *p*-value = 0.005). Similar results were found using the fixed effect model. Potential publication bias was identified by visual assessment of the funnel plot and confirmed by Egger’s linear regression test (intercept −0.72, *p*-value = 0.078). After applying the trim and fill method, the estimated effect size of the fixed effect was not significantly different from the main result, while the estimated effect size of the random effect was not statistically significant (Table 3). Again, an alternative analysis was performed using the two MedDiet scores calculated by Hosking et al. [45], but the results did not change.

A sensitivity analysis focusing only on unspecified dementia (excluding Alzheimer’s disease and mild cognitive impairment) was performed. In this analysis, 13 studies were included and using the random effects model, the pooled OR was 0.94 [(95% CI = 0.88–0.99); *p*-value = 0.005] based on 59,571 participants, with moderate statistical heterogeneity (df = 12, *I*^2^ = 59.86, *p*-value = 0.003). Similar results were found using the fixed effects model. No potential publication bias was detected by visual assessment of the funnel plot and confirmed by Egger’s linear regression test (intercept −0.62, *p*-value = 0.239) (Table 3).

A sensitivity analysis focusing on Alzheimer’ disease only was performed. In this analysis, eight studies were included and using the random effects model, the pooled OR was 0.73 [(95% CI = 0.62–0.85); *p*-value < 0.001] based on 38,292 participants, with moderate statistical heterogeneity (df = 7, *I*^2^ = 63.85, *p*-value = 0.007). Similar results were found using the fixed effects model. Potential publication bias was detected by visual assessment of the funnel plot and confirmed by Egger’s linear regression test (intercept −1.88, *p*-value = 0.055) (Table 3). After applying the trim and fill method, the estimated effect sizes for both fixed and random effects remain relatively consistent (Table 3).

A sensitivity analysis focusing on mild cognitive impairment only was performed. In this analysis, only three studies were included and using the random effect model, the pooled OR was 0.89 [(95% CI = 0.79–1.01); *p*-value = 0.063] based on 2996 participants, with low statistical heterogeneity (df = 2, *I*^2^ = 35.49, *p*-value = 0.212). A statistically significant result was found using the fixed effects model. Potential publication bias was identified by visual assessment of the funnel plot and confirmed by Egger’s linear regression test (intercept −2.72, *p*-value = 0.259). After applying the trim and fill method, the estimated effect size for both the fixed and random effects was no longer statistically significant (Table 3).

A sensitivity analysis based on methodological quality was not performed as all included studies were of high quality. Finally, studies based on the same population were excluded to avoid potential overlapping effects. In this analysis, only three studies were included and using the random effects model, the pooled OR was 0.89 [(95% CI = 0.82–0.97); *p*-value = 0.005] based on 58,813 participants, with moderate statistical heterogeneity (df = 15, *I*^2^ = 62.80, *p*-value < 0.001). Similar results were found using the fixed effects model. Potential publication bias was identified by visual assessment of the funnel plot and confirmed by Egger’s linear regression test (intercept −0.93, *p*-value = 0.126). After applying the trim and fill method, the estimated effect sizes remain similar for both fixed and random effects (Table 3).

### Subgroup analyses

A subgroup analysis by study design was performed for the main analysis (all types of dementia) and for each type of dementia. In particular, considering cross-sectional studies focusing on all types of dementia, seven studies were included and using the random effect model, the pooled OR was 0.91 [(95% CI = 0.82–1.00); *p*-value = 0.055] based on 10,029 participants, with moderate statistical heterogeneity (df = 7, *I*^2^ = 52.25, *p*-value = 0.050). More statistically significant results were found using the fixed effects model. No potential publication bias was identified by visual assessment of the funnel plot and confirmed by Egger’s linear regression test (intercept −0.78, *p*-value = 0.394) (Table). Focusing on cohort studies, 15 studies were included and using the random effect model, the pooled HR was 0.84 [(95% CI = 0.76–0.94); *p*-value = 0.002] based on 55,205 participants, with high statistical heterogeneity (df = 14, *I*^2^ = 89.70, *p*-value < 0.001). After applying the trim and fill method, the estimated effect size for both fixed and random effects did not change (Table 3).

Subgroup analyses by study design for dementia, unspecified dementia, Alzheimer’s disease and MCI are reported in Table 3. Subgroup analysis by geographical area was performed by grouping countries in the Mediterranean area. In this case, six studies were included and the pooled OR was 0.95 [(95% CI = 0.91–0.99); *p*-value < 0.001] based on 6879 participants, with low statistical heterogeneity (df = 5, *I*^2^ = 31.91, *p*-value = 0.196). Potential publication bias was identified by visual assessment of the funnel plot and confirmed by Egger’s linear regression test (intercept −0.86, *p*-value = 0.171). After applying the trim and fill method, the estimated effect size for both fixed and random effects did not change (Table 3).

Subgroup analysis by sex was not possible because less than three studies reported data for sex separately. Finally, as all the studies used validated tools to diagnose dementia, only a sensitivity analysis based on studies that used validated tools to assess diet was performed. In this case, 20 studies were included and the pooled OR was 0.89 [(95% CI = 0.83–0.94); *p*-value < 0.001] based on 37,930 participants, with moderate statistical heterogeneity (df = 19, *I*^2^ = 71.07, *p*-value < 0.001). After applying the trim and fill method, the estimated effect size of the fixed effect was not significantly different, while the estimated effect size of the random effect was not statistically significant (Table 3).

## Discussion

In this systematic review and meta-analysis, we assessed the association between the highest level of adherence to the MedDiet and the likelihood of developing dementia. The main results suggest that the highest adherence to the MedDiet is associated with an approximate 11% reduction in the likelihood of developing dementia in a population of 65,955 older adults. Despite the apparently small protective effect, it should be borne in mind that dementia is a frequently diagnosed disease, especially in the elderly. Furthermore, dementia has a high burden in terms of cost of care and quality of life. Furthermore, it is important to consider that the world's population is undergoing a progressive aging process, wherein the elderly constitute a significant proportion of the population. Therefore, even though the estimated effect size may be relatively modest, this applies to a rather large segment of the population, possibly on the rise. Additionally, it is crucial to bear in mind that the estimated effect in this meta-analysis is attributable solely to adherence to the MedDiet. This implies that by improving one's diet alone, the risk of developing dementia could be significantly reduced. Greater results could be achieved by implementing multiple healthy lifestyle choices [55]. Lastly, it should not be underestimated that this effect is linked to a primary prevention effect, the cost of which is negligible, especially when considering the high burden of dementia. Our results can be considered reliable insofar as we took into account several methodological aspects. First, we used both fixed effects and random effects models, the latter of which are recommended in the case of high to moderate statistical heterogeneity; however, the results did not change significantly. Secondly, given the heterogeneity of the MedDiet scores used in the original studies, we performed supplementary analyses using the MedDiet scores as an alternative, without this affecting the results. This type of analysis was performed primarily to test whether the use of different scales might affect the strength of the association. The same rationale was applied in the sensitivity analysis, for which we only considered studies that reported using a validated dietary assessment tool. In addition, as some studies were conducted based on the same population, only those with the largest sample size were included, thus eliminating the potential overlap effect. Even in this case, the results did not change significantly, confirming the robustness of our findings. To strengthen our results, we also estimated the risks separately by type of dementia, looking specifically at the risk of all types of dementia, all types of dementia without Alzheimer’s disease, dementia without Alzheimer’s disease and MCI, Alzheimer’s disease only, and MCI only. The association was found to be stronger only when only Alzheimer’s disease was considered, with the results suggesting that higher adherence to the MedDiet is associated with an approximately 27% lower risk of Alzheimer’s disease. On the contrary, the association was no longer significant when looking at MCI alone. However, only three studies reported separate data for MCI, so this result should be interpreted with caution. In fact, the sample size was relatively small, which may have affected the statistical power. Moreover, to assess the risk of prevalent and incident dementia separately, subgroup analyses were performed using only cross-sectional or cohort studies, respectively. When only cross-sectional studies were considered, the results remained relatively consistent for each of the type of dementia considered. Conversely, when only cohort studies were included, the association was borderline significant. This could be explained by the smaller sample size or by an inherent methodological weakness of cohort studies. In fact, longitudinal studies may be prone to selection bias, especially in the elderly population, who may be lost to follow-up for various reasons, including death. Moreover, specifically for dementia, it can be difficult or less accurate to assess exposure using questionnaires, thus affecting the certainty of results. From this perspective, case–control studies could also be a valid instrument for assessing the association between MedDiet and dementia, also considering that only one case–control study was retrieved and included in the current meta-analysis. Furthermore, dietary intake, by definition, is characterized by intrinsic methodological challenges. Dietary intake is measured and then quantified using self-reported data (questionnaires, diaries or 24-h recalls) which may be subject to recall bias, social desirability bias and even misreporting or misclassification. Moreover, dietary habits are culturally specific and assessing adherence to the MedDiet even in non-Mediterranean countries can be more complicated because certain Mediterranean foods are consumed less frequently or not at all, or, conversely, others may be consumed more frequently but not considered in the MedDiet scores, thus altering dietary intake. Moreover, due to the generally low adherence to the MedDiet, especially in non-Mediterranean countries, differences between groups can be more difficult to define, and the possible association between exposure and outcome(s) can be blurred. Therefore, we performed a subgroup analysis that only included studies conducted in the Mediterranean area. In this case, higher adherence to the MedDiet was associated with a 4–5% reduced risk of all types of dementia (considering fixed and random effects model, respectively). This risk reduction is lower than in the main analysis and when only Alzheimer’s disease was considered, probably because it included only six studies with a small sample size (*n* = 6879 subjects).

Lastly, it was not possible to analyse the subgroups by sex because fewer than three studies reported data for both sexes separately. In this respect, further research is needed to assess differences between the two sexes in elderly people. However, a previous meta-analysis assessing the association between MedDiet and cognitive health highlights attenuated results when only women were considered [56]; which potentially suggests that the cognitive effect of the MedDiet differs between sexes. Moreover, despite some differences in terms of inclusion/exclusion criteria, our results are similar to previously published meta-analyses, which mostly involve only cohort studies (usually with at least 1 year of follow-up) and including adults in general (not only the elderly, as in our case). In particular, the meta-analysis conducted by Cao et al. included only 4 studies and the estimated risk reduction was around 31% [RR = 0.69 (95% CI 0.57–0.84)] considering dementia or MCI [57]. The meta-analysis conducted by Singh et al. found a 33% risk reduction between the higher tertiles of MedDiet adherence and MCI or Alzheimer [HR = 0.67 (95% CI 0.55–0.81)], based on five studies [58]. Wu et al. also conducted a meta-analysis assessing the association between MedDiet and all types of dementia. This included nine cohort studies and the estimated risk reduction was 21% [RR = 0.79 (95% CI 0.70–0.90)], with no evidence of significant heterogeneity. However, the meta-analysis conducted by Coelho-Júnior et al. specifically focused on the elderly (adults over 60). However, despite finding a significant association between higher adherence to the MedDiet and multiple functional and cognitive functions (such as walking speed, knee muscle strength, global cognition and memory), they failed to find a significant association with all types of dementia (seven studies), Alzheimer’s (five studies) and MCI (three studies) [17].

### Potential biological mechanisms

Cardiovascular risk factors such as hypertension, obesity (mainly abdominal obesity), dyslipidaemia, and type 2 diabetes are considered to have a significant impact on the risk of dementia [59]. These factors are indeed associated with chronic inflammation and metabolic dysfunctions such as insulin resistance and consequent hyperinsulinemia that could be detrimental to the brain [60–62]. Findings from several studies have shown that a high adherence to the MedDiet can lead to a reduction in several biomarkers of inflammation known to be implicated in the onset of AD, such as pro-inflammatory cytokines interleukin-1β (IL-1β), interleukin-6 (IL-6), and tumour necrosis factor-alfa (TNF-α) [63–65]. The role of the MedDiet in reducing chronic inflammation seems to be mediated by the anti-oxidant and anti-inflammatory action of the numerous bioactive compounds such as vitamins, minerals, phytochemicals, and essential fatty acids, provided by foods typically consumed as part of this dietary pattern [66]. In addition, the lower energy density of the MedDiet, as compared to western dietary pattern, improves weight management and helps to reduce adipose tissue (especially abdominal fat) leading to a decrease in the production of pro-inflammatory cytokines, improved insulin-resistance and hyperinsulinemia, and improvements in other parameters such as hypertension and fasting glucose levels [67].

Moreover, there are other possible factors that could explain the positive effect of the MedDiet in preventing cognitive disorders. In particular, the MedDiet seems to help regulate the structure and function of the gut microbiota [68]. Dysbiotic gut microbiota are believed to play a role in the pathogenesis of cognitive decline, and in particular in AD, leading to synaptic dysfunction and neuroinflammation [69]. The MedDiet, in which is rich in fibre, plant protein and healthy fats (mainly from seafood, nuts and olive oil), along with a limited amount of saturated fat, animal protein and refined sugar, has been shown to have a positive impact on microbiota composition by increasing the bacteria that produce short-chain fatty acids, which are metabolites with anti-inflammatory effect [68]. Moreover, higher adherence to MedDiet has been associated with higher biodiversity of microbiota, which in turn seems to be associated with a regulation of cognitive functions [70]. In addition, a state of eubiosis has been shown to help reduce endothelial dysfunction, which is another known risk factor of cognitive [71].

### Implications for policy, practice and future research

Our data show an 11% reduction in the risk of all types of dementia and a 27% reduction in the risk of Alzheimer’s disease in people who follow the MedDiet. However, moderate heterogeneity was observed in the main analysis and in almost all sensitivity and subgroup analyses. Furthermore, when only cohort studies were considered individually, the statistical significance was found to be borderline or no longer significant. One interpretation of this finding could be that diet, and in particular stronger adherence to the Mediterranean diet, may have a greater protective effect against Alzheimer’s disease than against dementia in general. Although the underlying pathophysiological mechanisms of the protective role of the MedDiet against dementia are not clear, some studies found a significant correlation between increased adherence to the MedDiet and lower Alzheimer’s disease biomarker burden. Specifically, Hill et al. found that higher adherence to the MedDiet was associated with a lower deposition [0.11 (95% CI 0.04–0.17)] of beta-amyloid (Aβ), a key protein in the Alzheimer disease pathogenesis [72]. Furthermore, in another study, adherence to a MedDiet was found to be inversely correlated with brain positron emission tomography (PET) for both beta-amyloid plaques and tau tangles, meaning that higher adherence to the MedDiet is associated not only with lower deposition of beta-amyloid, but also with lower protein tau accumulation [73]. Consequently, higher adherence to the MedDiet could have a preventive impact on both the main pathogenetic pathways of Alzheimer's disease.

It is also important to bear in mind that our study population is made up of individuals over the age of 60. In such individuals, it is conceivable that the effects of recently adopted dietary habits may have less influence on health outcomes such as dementia (which require long-term exposure), as compared with dietary habits followed throughout their lifetime. However, the over-60s are the population most affected by dementia. It is, therefore, very important to consider diet as a potential exposure factor that can modify the risk of this disease, both in terms of prevention and healthcare policies, especially considering that there is still no direct treatment for dementia. Moreover, it should also consider that adherence to MedDiet is decreasing overtime, with the exception registered during COVID-19 pandemic [74]. These data prompt reflections in terms of public health. Specifically, if spending more time at home during the COVID-19 pandemic has led to increased adherence to the MedDiet [74], public health strategies should focus not only on greater nutrition education closely tied to the characteristics of the MedDiet itself [75, 76]; but also, on promoting policies that facilitate adherence to the MedDiet in all settings. In this regard, much is being done to facilitate the availability of healthy food options for consumption during meals outside the home [77]. However, more should be done to promote culinary skills that enable individuals to prepare healthy dishes [78, 79], as well as invest time in their preparation, possibly facilitating the sharing of these moments with friends and family (conviviality). These are essential elements of the MedDiet that, although supported by scientific knowledge, are not yet effectively integrated into health policies and campaigns for promoting and educating about nutrition and health. This applies to both the general population and specific target groups, as well as professionals in the food and health sector.

Like many other lifestyle recommendations, adopting a Mediterranean diet confers many additional health benefits, such as the positive effects on mental health, as well the associated reduced incidence of cardiovascular diseases and diabetes [80–86]. This means that the decision to implement health policies aimed at promoting this type of diet can be advantageous in the long term as it has positive repercussions on numerous health outcomes of public interest. Efforts should not only be directed towards changing the population's eating habits; the promotion of a healthy lifestyle must also be supported by much broader policies to make healthy choices easier. For example, adopting an effective food nutrition labelling system could help consumers make healthier food choices. Furthermore, the MedDiet has been shown to be a sustainable and cost-effective way of reducing the risk of many health conditions [87].

Lastly, starting from our results, future studies need to more deeply explore the underlying biological mechanisms through which the MedDiet may influence dementia, and therefore comparing the effectiveness of the MedDiet with other dietary patterns in reducing the risk of dementia. As well as, investigate the potential synergistic effects of combining adherence to the MedDiet with other healthy lifestyle factors, such as physical activity, mental stimulation, and social engagement still remain an important aspect to be further assessed in future. Lastly, scientific collaboration across countries might contribute in definition of a globally recognised method for assessing MedDiet adherence. This could reduce the high heterogeneity found regarding MedDiet scores and methods, improve comparisons among different regions, and foster scientific collaborations.

### Limitations and strengths

Our results should be interpreted with caution because they do involve some limitations: firstly, this is a secondary analysis (review of original studies) and, therefore, it is automatically influenced by the limitations of each of the included studies, such as potential selection bias or bias in exposure or outcome assessment. These limitations include the fact that dietary intake was self-reported, with potentially risk of recall or social desirability bias, and that several types of MedDiet scores were used. There was also high heterogeneity, probably because of the different types of scores used, or because of the different type of potential confounders considered in each included study; a third limitation is that most of the included studies were cross-sectional, in which causality might not be assessed by definition.

Nonetheless, the current systematic review has certain strengths: first, we followed the PRISMA guidelines which allow us to use a comprehensive approach both for conduct and reporting; we also consulted three different databases in order to retrieve all eligible studies (more than the minimum required by guidelines). We conducted multiple sensitivity and subgroup analyses to assess the association between several types of dementia, as well as the study design, geographical area, or MedDiet score used. In contrast with previous meta-analysis, we did not exclude cross-sectional studies, which are a valuable study design especially when cohort studies are difficult to perform. As mentioned before, longitudinal studies among the elderly might be biased because of the potentially high number of lost to follow-up. Moreover, even if in cross-sectional studies exposure and outcome are measured at the same time-point, it is challenging to consider that a higher adherence to MedDiet has occurred due to dementia.

## Conclusions

In conclusion, the present study assessed the association between adherence to the Mediterranean diet and all types of dementia (stratifying the results by type of diagnosis) in the elderly population aged over 60 years. There is a protective effect of the Mediterranean diet when all types of dementia are considered together and when only Alzheimer's disease is considered individually. Specifically, there is an 11% reduction in the risk of all types of dementia and a 27% reduction if only Alzheimer's disease is considered. Given the moderate heterogeneity observed and the limitations mentioned above, these results should be interpreted with caution. However, even if the risk reduction is minimal, especially when all types of dementia are considered, it is true that it affects a relatively large number of people, especially the elderly. Therefore, even a small percentage reduction would represent a significant number of people who could potentially prevent dementia just by increasing their adherence to the Mediterranean diet. Consequently, our results confirm the importance of promoting adherence to the Mediterranean diet in order to improve cognitive health in aging populations.

### Supplementary Information

Below is the link to the electronic supplementary material.

Supplementary file1 (DOCX 22 KB)

## Data Availability

Not applicable.
