# Supplementary material for: Association between Mediterranean diet and dementia and Alzheimer disease: a systematic review with meta-analysis
Source: Aging Clin Exp Res. 2024 Mar 22;36(1):77. doi: 10.1007/s40520-024-02718-6 (PMC10959819; doi:10.1007/s40520-024-02718-6)
Supplement: Supplementary file 1 — Supplementary file1 (DOCX 22 KB) [file 40520_2024_2718_MOESM1_ESM.docx]

**Supplementary Table 1: Search strategy, for each databased**

| **Database** | **Search Strategy** | **Number of retrieved records** |
| --- | --- | --- |
| PubMed/MEDLINE | ((("score*"[Title/Abstract] OR "pattern*"[Title/Abstract] OR "index*"[Title/Abstract] OR "adherence"[Title/Abstract] OR "behaviour*"[Title/Abstract] OR "behavior*"[Title/Abstract] OR "diet*"[Title/Abstract] OR "intake"[Title/Abstract]) AND "mediterranean"[Title/Abstract]) OR "diet, mediterranean"[MeSH Terms]) AND ("amentia*"[Title/Abstract] OR "dementia*"[Title/Abstract] OR "alzheimer*"[Title/Abstract] OR "Alzheimer Disease"[MeSH Terms] OR "Dementia"[MeSH Terms]) AND ("Aged"[MeSH Terms] OR "Aged"[Title/Abstract] OR "senile"[Title/Abstract] OR "old"[Title/Abstract] OR "elder*"[Title/Abstract]) | 257 |
| Scopus | ( ( TITLE-ABS-KEY ( mediterranean ) ) AND ( TITLE-ABS-KEY (index* OR score* OR pattern* OR adherence OR behaviour* OR behavior* OR diet* ) ) ) AND ( TITLE-ABS-KEY ( amentia OR dementia OR alzheimer* ) ) AND ( TITLE-ABS-KEY ( elder* OR old OR senile OR aged ) ) | 425 |

**Supplementary Table 2: Detailed inclusion/exclusion criteria, defined according to PECOS (Population, exposure, comparison, outcome, study design).**

| **Search Strategy** | **Details** |
| --- | --- |
| **Inclusion criteria** | P: women and men aged equal or more than 60 years  E: highest adherence to Mediterranean diet estimated using any type of score  C: lowest adherence to Mediterranean diet, or no adherence  O: Risk of any type of dementia, including mild cognitive impairment (assessed by neurological tests, and/or medical records, and/or clinical/neurological visit/examination, and/or neurological imaging, and/or blood tests)  S: original, observational study (including cross-sectional, case-control, or cohort both prospective and retrospective studies), published as peer-reviewed articles in international scientific journals |
| **Exclusion criteria** | P: women and men younger than 60 years old  E: adherence to diet different than Mediterranean diet, or a combination of multiple diets, or intake of single food components, or supplementation  C: other type of diet  O: other health outcomes  S: not original (reviews with or without meta-analysis), not performed among humans, not observational (as for instance trials), not published as peer-reviewed articles in international scientific journals (book, book chapter, thesis), no full-text papers (abstract, conference paper, letter, commentary, note) |
| **Language** | English |
| **Time filter** | none |

**Supplementary Table 3: Articles excluded with reasons**

| **Reasons** | **Number of studies** | **References** |
| --- | --- | --- |
| Younger population | 6 | Andreu-Reinón, M.E.; 2021  Cornelis, M. C.; 2022  Filippini, 2020  Hu, E. A., 2020  Nezire Ince, 2020  Takeuchi, 2021 |
| Not Mediterranean diet alone, but in combination (as for instance MIND diet), or in combination with other lifestyle (as for instance physical activity) | 3 | Dhana, K. 2020  Thomas, 2022  Chen H |
| Different outcome | 2 | S.A. White, 2020  Scarmeas, 2007 |

**Supplementary Table 4. Item-by-item quality assessment of each included studies, reported in alphabetical order**

| **Author, year** | **Item 1** | **Item 2** | **Item 3** | **Item 4** | **Item 5a** | **Item 5b** | **Item 6** | **Item 7** | **Item 8** | **Total score** | **Quality** | **Conflicts of interest** | **Funds** |
| --- | --- | --- | --- | --- | --- | --- | --- | --- | --- | --- | --- | --- | --- |
| Allcock, 2022 | * | - | * | * | * | * | ** | * | - | 8 | High | no | yes |
| Chan, 2013 | * | - | * | * | * | * | ** | * | - | 8 | High | no | yes |
| Charisis, 2021 | * | * | * | * | * | * | * | - | * | 8 | High | no | yes |
| Anastasiou, 2017 | * | - | - | * | * | * | ** | * | - | 7 | High | no | yes |
| Calil | * | - | * | * | - | - | ** | - | - | 5 | Moderate | no | yes |
| de Crom, 2022 | * | * | * | * | * | * | * | * | * | 9 | High | yes | yes |
| Féart, 2009 | * | * | * | * | * | * | * | * | * | 9 | High | yes | no |
| Gardener, 2012 | * | - | * | * | * | * | ** | * | - | 8 | High | no | yes |
| Glans, 2023 | * | * | * | * | * | * | * | * | * | 9 | High | yes | yes |
| Gu, 2010 | * | * | * | * | * | * | * | * | - | 8 | High | n.a. | n.a. |
| Haring, 2016 | * | * | * | * | * | * | * | * | * | 9 | High | n.a. | yes |
| Hosking, 2019 | * | * | * | * | * | * | * | * | - | 8 | High | no | yes |
| Mamalaki, 2022 | * | * | * | * | * | - | * | - | * | 7 | High | no | yes |
| Morris, 2015 | * | * | * | * | * | * | * | * | * | 9 | High | no | yes |
| Roberts, 2010 | * | * | * | * | * | * | * | - | - | 7 | High | no | yes |
| Nicoli, 2021 | * | - | * | * | * | * | ** | * | - | 8 | High | no | yes |
| Olsson, 2014 | * | * | * | * | * | * | - | * | * | 8 | High | yes | n.a. |
| Scarmeas, 2006 (a) | * | - | * | * | * | * | * | * | - | 7 | High | n.a. | yes |
| Scarmeas, 2006 (b) | * | * | * | * | * | * | * | - | * | 8 | High | n.a. | n.a. |
| Scarmeas, 2009 | * | * | * | * | * | * | * | * | * | 9 | High | n.a. | n.a. |
| Talhaoui, 2023 | * | - | - | * | * | * | ** | * | - | 7 | High | no | no |
